# Down-regulation of EDN1 gene expression by circulating miR-206 is associated with risk of preeclampsia

**DOI:** 10.1097/MD.0000000000020319

**Published:** 2020-05-29

**Authors:** Chunzhi Sheng, Yangchun Zhao, Libo Zhu

**Affiliations:** a Department of obstetrics and gynecology, Wenling Hospital of Traditional Chinese Medicine; b Department of Obstetrics and Gynecology, the Second Affiliated Hospital of Zhejiang Chinese Medical University; c Department of Gynaecology and Obstetrics, Affiliated Hangzhou First People's Hospital, Zhejiang University School of Medicine, Zhejiang, PR China.

**Keywords:** correlation, endothelin-1, microRNA-206, preeclampsia

## Abstract

To study the correlation between circulating microRNA-206 (miR-206) levels and endothelin-1 (ET-1) levels, and to explore its association with preeclampsia (PE) risk.

Reverse transcription-PCR (RT-PCT) was used to compare the plasma miR-206 levels in 200 PE patients and 200 healthy controls. The correlation between miR-206 and ET-1 levels in plasma of PE patients was analyzed by Pearson analysis. MiR-206 was transfected into human umbilical vein endothelial cells cells and ET-1 expression was analyzed by enzyme-linked immunosorbent assay.

RT-PCR results showed that plasma miR-206 levels in PE patients were significantly higher than those in the control group (*P* < .01). The results of receiver operating characteristic curve analysis showed that the area under the curve of plasma miR-206 level in the diagnosis of PE was 0.94 (95% confidence interval: 0.92–0.96). Plasma ET-1 levels in PE patients were significantly lower than those in the control group by enzyme-linked immunosorbent assay (*P* < .01). The area under the curve of plasma ET-1 level in the diagnosis of PE was 0.92 (95% confidence interval: 0.90–0.95). The level of miR-206 in plasma was negative correlated with ET-1 level (*r* = -0.37, *P* < .01). The expression level of ET-1 was significantly decreased in human umbilical vein endothelial cells cells transfected with miR-206.

miR-206 can down-regulate the expression of EDN1 gene, which may be related to the increased risk of preeclampsia.

## 1 Introduction

Preeclampsia (PE) is a serious pregnancy condition characterized by new-onset hypertension, proteinuria and other typical signs that can negatively affect the development of pregnancy. The disorder can progress to multi-organ dysfunction, including hepatic, renal, and cerebral disease that is associated with substantial maternal and fetal morbidity and mortality.^[1,2]^ The causative factors and pathophysiological mechanisms of PE are still unclear, however, it is generally believed that placental dysfunction is the main cause of PE, as this complex pathophysiology is ameliorated after delivery.^[3–7]^

MicroRNAs (miRNAs) are small (∼22–25 nt), endogenous, single-stranded, non-coding RNAs involved in post-transcriptional gene silencing by binding to the untranslated region (3′UTR) of a target gene. They play significant roles in many pathological and physiological processes, such as cellular growth, development, organogenesis, and apoptosis. In recent years, studies have shown differential expression of miRNA in pregnant patients with PE, suggesting that miRNAs may be important regulatory molecules in the development of PE.^[8]^

MiR-206 is 1 of the most studied and best characterized miRNA to date, which specifically expressed in skeletal muscle.^[9]^ Numerous studies have shown that miR-206 is a key modulator of skeletal muscle development and disease. Importantly, dysregulation of miR-206 has been linked to cancer progression, neurological diseases and myocardial infarction.^[10–12]^ Recently, miR-206 was identified as a novel factor upregulated in PE within the maternal circulation and in placental tissue.^[13]^ To reveal the function of miR-206 in PE patients, the online TargetScan (http://www.targetscan.org/mamm_31/) was used to predict possible miR-206 sites. MiR-206 binding site sequences in the 3′UTR region of EDN1 gene was identified.

Recently it was suggested that abnormal endothelial function may contribute to the pathophysiological changes observed in PE. Endothelin-1 (ET-1) was vasoactive substances produced by endothelial cells and an association between an ET-1 gene polymorphism with high ET-1 and PE was reported in humans, despite their cause and effect relationships have not been defined.^[14,15]^ There is literature reported that the paternal rs5370 SNP was associated with a reduced risk of PE.^[14]^ Maternal ET-1 was found to be elevated in PE patients and correlated with the severity of blood pressure elevation.^[14]^ The ET-1 is likely released from the maternal endothelium. Presence of T allele at 5665 locus in maternal EDN1 gene is associated with higher ET-1 levels.^[15]^ Recent literature suggests that TNF-α-induced hypertension was associated with significant increases in renal and placental ET-1.^[16]^ Additionally, ET-1 may be 1 of the key links between primary placental disorders and the systemic endothelial dysfunction of PE.^[17]^ This study was performed to obtain a comprehensive estimate of the putative influence of miR-206 on EDN1 gene expression, further determining its association with PE and the underlying molecular mechanisms.

## 2 Materials and methods

### 2.1 Patient

From August 2014 to August 2018, a total of 200 consecutive patients (31.2 ± 4.8 years) with a primary diagnosis of PE were recruited from the Department of Obstetrics and Gynecology, Tongde Hospital of Zhejiang Province. Meanwhile, 200 healthy pregnant women were consecutively enrolled as the control group. The diagnosis of PE was made in consistent with the criteria established by the American College of Obstetricians and Gynecologists, currently meeting “a and b” as followings: a. Blood pressure:

(1) greater than or equal to 140 mm Hg systolic or greater than or equal to 90 mm Hg diastolic on 2 occasions at least 4 hours apart after 20 weeks of gestation in a woman with a previously normal blood pressure.
(2) greater than or equal to 160 mm Hg systolic or greater than or equal to 110 mm Hg diastolic, hypertension can be confirmed within a short interval (minutes) to facilitate timely antihypertensive therapy.

b. Proteinuria:

(1) greater than or equal to 300 mg per 24-hour urine collection (or this amount extrapolated from a timed collection) or protein/creatinine ratio greater than or equal to 0.3 mg/dL;
(2) dipstick reading of 1+(used only if other quantitative methods not available).^[18]^

All cases were singleton pregnancies, and there were no other maternal complications such as multiple pregnancies, fetal anomalies, premature rupture of membranes, maternal infection, previous history of hypertension and/or cardiac or renal disease, diabetes, or smoking. This study was approved by the Ethics Committee of Hospital (No 2015031402P) and was carried out in accordance with the World Medical Association Declaration of Helsinki. Written, informed consents were obtained from all the patients (or their legal representatives) enrolled in this study.

### 2.2 Real-time PCR for miR-206

Total miRNAs was extracted from each plasma using miRNeasy Mini Kit (Cat. #217004, Qiagen). After quantification, cDNA was synthesized from 1 μg RNA by TaqMan MicroRNA Reverse Transcription Kit (Applied Biosystems, Foster City. Real-time PCR was performed by SYBR^[]^ Premix Ex Taq kit (Roche, Germany), U6 small nuclear RNA was used as internal control to normalize the expression of miR-206. The sequences of the miR-206 primers: forward: 5′-GGC GGT GGA ATG TAA GGA AG-3′; reverse: 5′-GGC TGT CGT GGA CTG CG-3′. U6 primers: forward: 5′-TGA CAC GCA AAT TCG TGA AGC GTT C-3′; reverse: 5′-CCA GTC TCA GGG TCC GAG GTA TTC-3′. The procedure of real-time PCR: initialization through a temperature of 95°C for 30 seconds; denaturation through a temperature of 95°C for 5 seconds; annealing through a temperature of 60°C for 34 seconds; 40 cycles. The formula RQ=2^-ΔΔCT^ was used to calculate the relative gene expression after normalization with internal controls. Each sample had at least 3 triplicates.

### 2.3 Enzyme-linked immunosorbent assay (ELISA) for ET-1

Each participant donated a 4 ml blood sample from ulnar vein. Human umbilical vein endothelial cells (HUVECs) cell supernatant were collected. Plasma were extracted from each whole blood after centrifugation at 2000 g for 20 minutes and then stored at -80°C. Soluble ET-1 were quantified using an ELISA kit (catalog no. QET00B; R&D Systems, Minneapolis, MN) according to the manufacturer's instructions. Each experiments represented at least 3 triplicates. The standard curve method was used to determine the ET-1 expression level. The sensitivity of this method was 0.10 ng/mL.

### 2.4 Transfection of miR-206 mimic and inhibitor

miRNA-206 mimic, inhibitor and the negative control were synthesized in Genepharma Co., Ltd. (Shanghai, China). Human umbilical vein endothelial (HUVEC) cells (2 × 10^5^) were seeded onto 6-well plates. The miRNA-206 mimic, inhibitor and the negative control were transfected in 80% confluent HUVEC cells using lipofectamine3000 (Invitrogen) according to the manufacturer's instructions, followed by incubation at 37°C and 5% CO_2_ overnight, after which the supernatant was removed for ET-1 measurement (ELISA) and the cells were collected for RNA isolation using Trizol reagent (Molecular Research Center, Cincinnati, Ohio). The sequences were shown as followings: miR-206 mimic (5′-UGG AAG UAA GGA AGU GUG UGG-3′), miR-206 inhibitor (5′-CCA CAC ACU UCC UUA CAU UCC A-3′) and miRNA negative control (5′- CAG UAC UUU UGU GUA GUA CAA-3′).

### 2.5 Real-time PCR for EDN1 measurement

Total miRNAs of HUVEC cells were isolated using miRNeasy Mini Kit (Qiagen, Leusden, Netherlands). High capacity cDNA reverse transcription with RNase inhibitor kit (Applied Biosystems-Life Technologies, Carlsbad, CA) was used to obtain cDNA. Real-time PCR was performed as above to measure EDN1 mRNA. The primers for EDN1: forward: 5′-CAA GCA GGA AAA GAA CTC AG-3′; reverse: 5′-CTG GTT TGT CTT AGG TGT TC-3′. β-actin acted as the internal control: forward: 5′-GGG AAT TCA AAA CTG GAA CGG TGA AGG-3′; reverse: 5′-GGA AGC TTA TCA AAG TCC TCG GCC ACA-3′. The formula RQ=2^-ΔΔCT^ was used to calculate the relative gene expression. Each sample had at least 3 triplicates.

### 2.6 Western blotting

HUVEC cells were lysed using RIPA buffer (Sigma-Aldrich). The supernatant was removed by centrifugation at 14000 rpm for 10 min and its totalprotein level was measured using bicinchoninic acid assay (BCA) kit (Pierce, Rockford, IL). Lysates were electrophoretically separated on a 4% to 15% gradient SDS-PAGE gel (Bio-Rad Laboratories, USA) and transferred to a nitrocellulose membrane. Subsequent procedures were modified from the standard protocol.

### 2.7 Dual luciferase activity assay

Predication of miR-206 target genes was carried out using TargetScan (http://www.targetscan.org/mamm_31/). Dual luciferase activity assay was used to assess the target of miR-206. MiR-206 binding site sequences in the 3′UTR region of EDN1 gene was identified. The wide type or mutant 3′UTR seed region of EDN1 was synthesized and cloned into a pMIR-REPORT luciferase vector (Invitrogen). HEK-293T cells were seeded into 24-well plates, and a hsa-miR-206 mimic, hsa-miR-206 inhibitor or control vector were co-transfected with the pMIR-REPORT luciferase vector containing firefly luciferase reporter gene using Lipofectamine 2000 (Invitrogen). After 48 hours of transfection, luciferase activity was analyzed using the dual luciferase assay system, according to the instruction book. Each experiment had at least 3 triplicates.

### 2.8 Statistical analysis

Data are presented as the mean ± SD or SEM from at least 3 independent experiments. Statistical analyses involved unpaired *t*-test, *χ*^*2*^ and Pearson correlation. GraphPad Prism 5.0 was used for the statistical calculations. *p* < .05 were considered statistically significant.

## 3 Result

### 3.1 Population characteristics

The baseline clinical characteristics of the enrolled patients and healthy controls were shown in Table 1. As expected, systolic and diastolic blood pressures were higher in the PE group when compared with the control group (*P* < .01). Except for weight at birth and family history of PE, there was no statistically significant differences in age, gestational age, body mass index and delivery mode between 2 groups (*P* > .05).

**Table 1:**
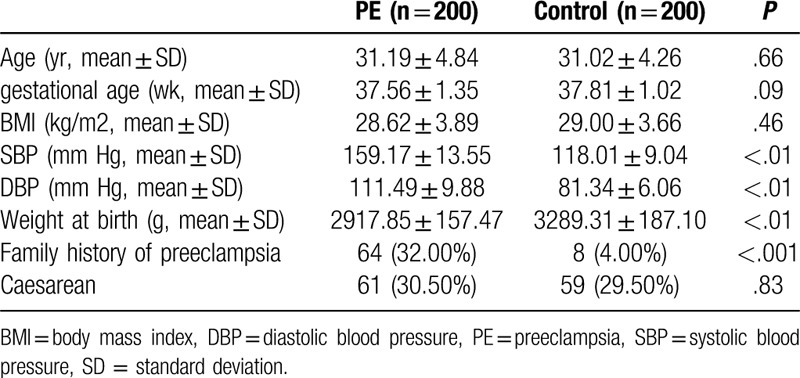
Comparisons of baseline characteristics between the case group and the control group.

### 3.2 Plasma expression of miR-206

**Figure 1:**
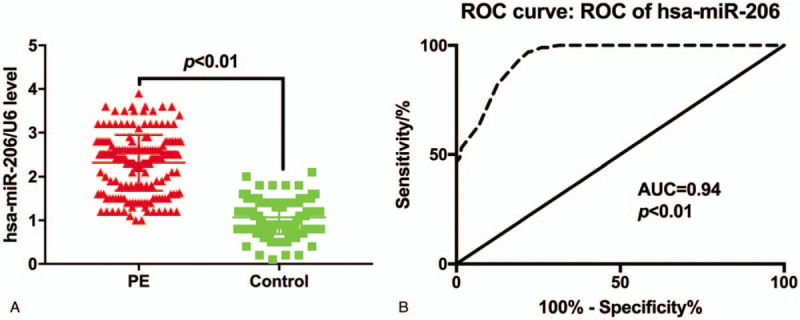
(A) Plasma levels of miR-206 in the case and control group; (B) ROC curve of miR-206. ROC = receiver operating characteristic.

Measurement of miR-206 expression level showed that the expression of miR-206 was significantly higher in PE patients (2.31 ± 0.64) in comparison with control group (1.07 ± 0.38, *P* < .01, Fig. 1A). The results of receiver operating characteristic curve analysis showed that the area under the curve of plasma miR-206 level in the diagnosis of PE was 0.94 (95% CI: 0.92–0.96), the cut-off value was 1.72, with the sensitivity of 97.00% (95% CI: 93.58%-98.89%) and specificity of 77.50% (95%CI: 71.08%-83.09%) (Fig. 1B).

### 3.3 Plasma expression of ET-1

**Figure 2:**
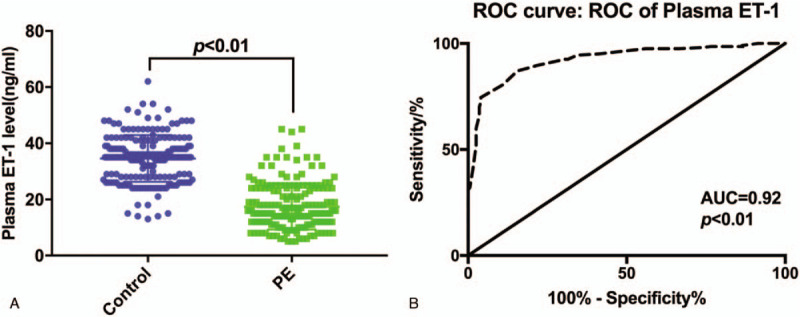
(A) Plasma levels of ET-1 in the case and control group; (B) ROC curve of ET-1. ROC = receiver operating characteristic.

Plasma ET-1 levels in PE patients (17.40 ± 8.11 ng/ml) were significantly lower than those in the control group (34.60 ± 8.28 ng/ml) by ELISA (*P* < .01, Fig. 2A) . The area under the curve of plasma ET-1 level in the diagnosis of PE was 0.92 (95% CI: 0.90–0.95), the cut-off value was 25.50 ng/mL, with the sensitivity of 87.00% (95%CI: 81.53%-91.33%) and specificity of 84.50% (95%CI: 78.73%-89.22%) (Fig. 2B).

### 3.4 Correlation between plasma miR-206 and ET-1

**Figure 3:**
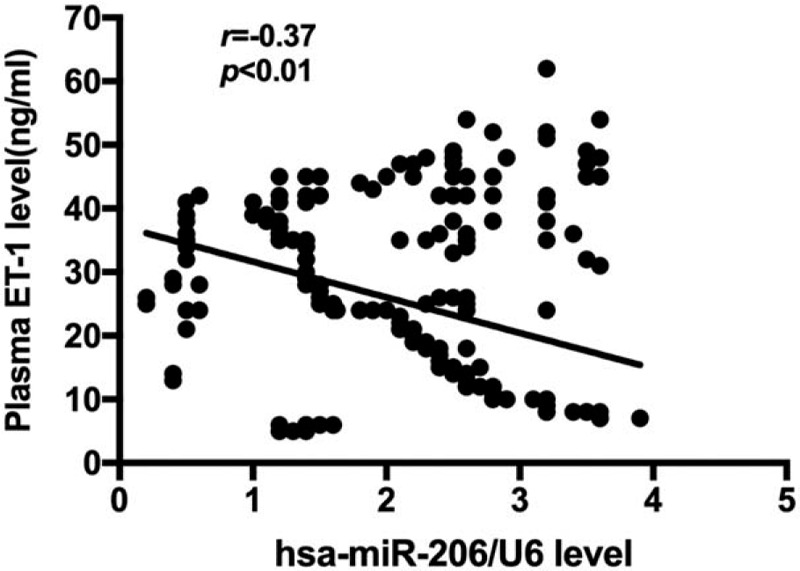
Correlation between the expression levels of ET-1 and miR-206 in the plasma. ET-1 = endothelin-1.

We further evaluated whether the miR-206 expression correlated with ET-1 expression. The Pearson correlation analysis identified a moderate negative correlation between miR-206 and ET-1 in the plasma of PE patients (*r* = -0.37, *P* < .01, Fig. 3).

### 3.5 miR-206 regulated ET-1 expression at the transcriptional level

**Figure 4:**
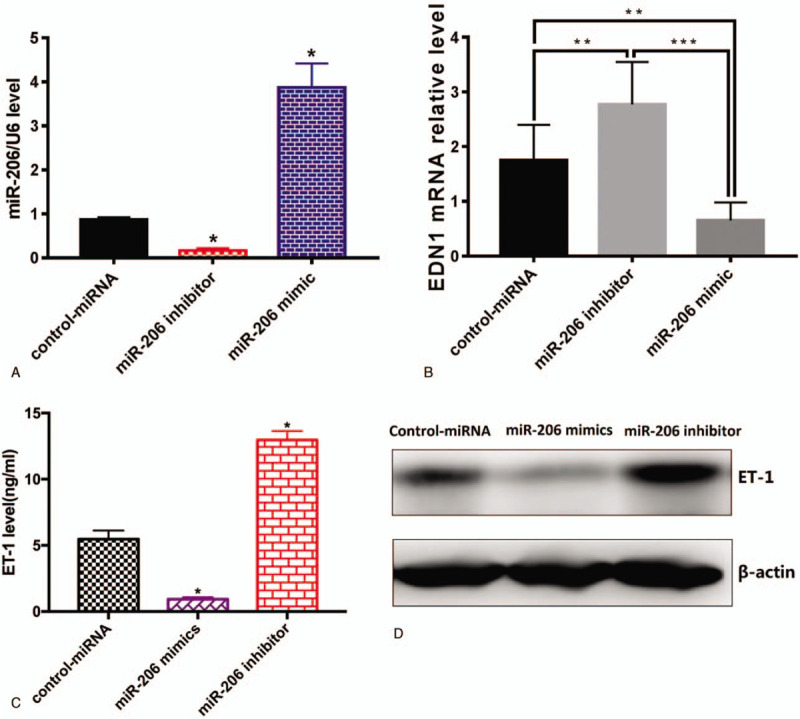
miR-206 increased EDN1 expression in HUVEC cells. HUVEC cells were transfected with miR-206 mimic, inhibitor and their negative controls. (A) miR-206 expression was assessed by RT-PCR. EDN1 levels were assessed by (B) real-time PCR (C) ELISA as well as (D) western blotting. EDN1 = endothelin 1 gene, HUVECs = human umbilical vein endothelial cells.

In order to change the levels of miR-206, HUVEC cells were transfected with miR-206 mimic, miR-206 inhibitor and the negative control miRNA. Real-time PCR experimental results showed the transient transfection of miR-206 mimic significantly up-regulated miR-206 expression in HUVECs cells, whereas transient transfection of miR-206 inhibitor down-regulate the expression of HUVECs cells (*P* < .01, Fig. 4A). Induction or inhibition of miR-206 using miR-206 mimic or inhibitor significantly reduced or increased the mRNA expression levels of EDN1 compared to the controls (*P* < .05, Fig. 4B). Western blotting was performed to evaluate whether miR-206 is involved in the regulation of ET-1 protein expression. Upregulation or downregulation of miR-206 through miR-206 mimic or inhibitor resulted in significantly suppression or induction of ET-1 expression (*P* < .05, Fig. 4C/D). These results indicated that miR-206 can down-regulate the expression of ET-1.

### 3.6 EDN1 was identified as a target of hsa-miR-206

**Figure 5:**
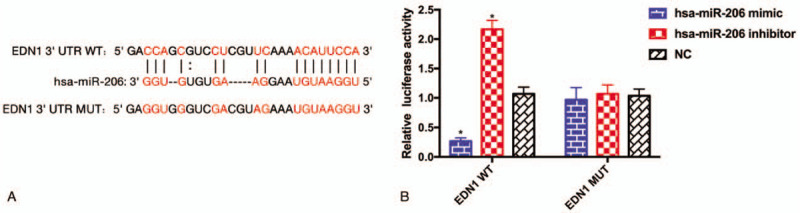
(A) The TargetScan was used to predict possible miR-206 binding site. (B) Dual luciferase activity assay was used to assess the target of miR-206. NC: no template control.

The TargetScan was used to predict the target gene of miR-206. ET-1 is encoded by EDN1 gene. In the 3′UTR region of EDN1, we have identified miR-206 binding site sequences (Fig. 5A), suggesting that ET-1 might be a potential target of miR-206. To verify the assumption, dual-luciferase assay was performed. Luciferase reporter gene assay suggested the EDN1-WT luciferase activity was obviously suppressed after co-transfection with of hsa-miR-206 mimic and significantly increased after co-transfection with of hsa-miR-206 inhibitor. However, there was no statistical difference in the EDN1-Mut luciferase activity after induction or inhibition of miR-206 (Fig. 5B). This finding suggested that EDN1 was the target gene of miR-206.

## 4 Discussion

In the present study, we found that miR-206 was significantly increased in the PE patients’ plasma, while ET-1 was significantly reduced in the patients with PE. Correlation analysis revealed that the miR-206 level was negatively correlated with ET-1 in PE patients. ET-1 was coded by EDN1. Mechanistically, bioinformatics analysis and dual luciferase reporter assay confirmed that EDN1 was the target gene of miR-206. These findings indicated that miR-206 may lead to an increased risk of PE via down-regulation of ET-1, though which needs to be further confirmed in vivo.

PE is a clinical syndrome occurring after 20 weeks gestation that affects an estimated 4% to 5% of pregnancies worldwide and is a major contributor to maternal and fetal morbidity and mortality worldwide.^[19,20]^ The etiology of PE has not yet been fully elucidated, the leading hypotheses strongly rely on disturbed placental function in early pregnancy as PE occurs only in the presence of placenta and its resolution begins after delivery. Therefore, the greatest focus on the pathogenesis of PE been on the placental dysfunction. However, genetic factors, immune factors, and systemic inflammation have also been confirmed to play a role in its pathogenesis and intense research efforts are now focused on PE to elucidate the exact pathophysiology.^[21]^ There is growing evidence for the involvement of many miRNAs in the fundamental biological processes, such as cell growth, organ development and tumorigenesis.^[22]^ Specific patterns of miRNA have been detected in the placenta, circulation, decidual-derived mesenchymal stem cells, umbilical cord blood, and human vein endothelial cells. Studies have established a close link between the disrupted expression of miRNAs and adverse pregnancy complications and multiple miRNAs were involved in placental development.^[23]^

MiR-206 is located on the human chromosome 6p12.2, which is essential for growth and rebuilding of skeletal muscle.^[24]^ It also plays an important role in tumor cell apoptosis, proliferation, migration, invasion, angiogenesis, drug resistance and cancer development. Thus far, miR-206 is mainly studied in malignancies, limited research has addressed the role of miR-206 in hypertension.^[25–28]^ A previous study in rat model has shown that knockdown of miR-206 significantly improved cardiopulmonary function and structure and rescued preexisting severe pulmonary hypertension, indicating that miR-206 might be a potential therapeutic target for hypertension.^[24]^ Our study revealed that plasma miR-206 was significantly higher in PE patients in comparison with healthy subjects, which is consistent with a previous finding that miR-206 is a novel factor up-regulated in PE within the maternal circulation and in placental tissue. ^[15]^ The molecular mechanism by which the miR-206 was up-regulated in PE patients remains to be fully elucidated.

While the exact causes of PE remain unknown, it is becoming increasingly evident that an important final common pathway whereby many soluble placental factors elicit cardiovascular and renal dysfunction during PE is by activation the endothelin (ET) system.^[29,30]^ It is now widely accepted that ET-1 plays a critical role in the pathophysiology of PE.^[31–35]^ Multiple studies report that ET-1 is increased in PE and some studies report a positive correlation between ET-1 and the severity of symptoms, though this is not a universal finding. ET-1 was coded by EDN1. Using dual luciferase reporter assay, we confirmed that EDN1 was the target gene of miR-206. As expected, we identified a moderate negative correlation between miR-206 and ET-1 in the plasma of PE patients. To further investigate whether miR-206 can regulate ET-1 expression, we performed the over-expression and interruption assay via adding miR-206 mimic and inhibitor. Results revealed that upregulation or downregulation of miR-206 resulted in significantly suppression or induction of ET-1 expression, suggesting an essential role of miR-206 for mediating ET-1 regulation.

Several limitations should be considered while interpreting the result of this study. First, we verified the regulatory role of miR-206 in HUVEC cells. As the biological function of a single miRNA could be cell-type specific, the exact role of miR-206 should be further explored in other cell types and in vivo. Additionally, we did not examine the expression of miR-206 and ET-1 in placenta tissue, which was far to enough to elucidate the critical role in pathogenesis. Last but not least, there are many other miRNAs that are closely linked to the susceptibility to PE, it might be interesting to explore whether the interactive role of miRNAs in PE development.

## 5 Conclusion

In the present study, we found that miR-206 was significantly increased in the PE patients’ plasma, while ET-1 was significantly reduced in the patients with PE. MiR-206 can down-regulate the expression of EDN1 gene, which may be related to the increased risk of PE.

## Author contributions

Libo Zhu conceived the idea, designed research and revised the article. Chunzhi Sheng and Yangchun Zhao collected the samples and performed most of the experiments and statistical analysis; Chunzhi Sheng drafted and revised the article. Chunzhi Sheng performed the statistical analyses. All authors read and approved the final manuscript.

**Conceptualization:** Libo Zhu.

**Data curation:** Chunzhi Sheng.

**Investigation:** Yangchun Zhao.

**Project administration:** Libo Zhu.

**Supervision:** Yangchun Zhao, Libo Zhu.

**Writing – review & editing:** Chunzhi Sheng.
